# Retraction Note: The best location for the application of static magnetic fields based on biokinetic coefficients in complete-mix activated sludge process

**DOI:** 10.1038/s41598-025-33110-7

**Published:** 2026-01-07

**Authors:** Ghorban Asgari, Abdolmotaleb Seid-Mohammadi, Reza Shokoohi, Mohammad Reza Samarghandi, Glen T. Diger, Behrooz Malekolkalami, Ramin Khoshniyat

**Affiliations:** 1 Social Determinants of Health Research Center (SDHRC), Faculty of Public Health, Department of Environmental Health Engineering, Hamadan University of Medical Sciences, Hamadan, Iran; 2 Department of Environmental Health Engineering, School of Public Health, Research Centre for Health Sciences, Hamadan University of Medical Sciences, Hamadan, Iran; 3 Department of Civil and Environmental Engineering, University of Michigan, 177 EWRE Building, 1351 Beal Street, Ann Arbor, MI 48109 USA; 4 Department of Physics, University of Kurdistan, Sanandaj, 66177-15175 Iran

Retraction of: *Scientific Reports* 10.1038/s41598-023-32285-1, published online 29 March 2023

The Editors have retracted this Article as it contains irrelevant references for which the Authors were unable to provide a suitable explanation. An investigation by the Editors determined that the authorship of this Article could not be fully verified. In addition, the Editors assessed the source data provided by the Authors but determined that data corresponding to Figures 2 and 3 was missing. The Editors no longer have confidence in the reliability of this Article.

Glen T. Daigger, whose name is incorrectly spelled as Glen T. Diger in the author list, agrees with this retraction and its wording. Ramin Khoshniyat disagrees with this retraction. Ghorban Asgari, Abdolmotaleb Seid-Mohammadi, Reza Shokoohi, Mohammad Reza Samarghandi, and Behrooz Malekolkalami did not respond to the Editors’ correspondence about this retraction.

